# Radiomics and AI-Based Prediction of MGMT Methylation Status in Glioblastoma Using Multiparametric MRI: A Hybrid Feature Weighting Approach

**DOI:** 10.3390/diagnostics15101292

**Published:** 2025-05-21

**Authors:** Erdal Tasci, Ying Zhuge, Longze Zhang, Holly Ning, Jason Y. Cheng, Robert W. Miller, Kevin Camphausen, Andra V. Krauze

**Affiliations:** Radiation Oncology Branch, Center for Cancer Research, National Cancer Institute, National Institutes of Health, 9000 Rockville Pike, Building 10, CRC, Bethesda, MD 20892, USA; erdal.tasci@nih.gov (E.T.); zhugey@mail.nih.gov (Y.Z.); longze.zhang@nih.gov (L.Z.); hning@mail.nih.gov (H.N.); jason.cheng@nih.gov (J.Y.C.); rwmiller@mail.nih.gov (R.W.M.); camphauk@mail.nih.gov (K.C.)

**Keywords:** MGMT, radiomics, image processing, machine learning, feature extraction, feature selection

## Abstract

**Background/Objectives**: Glioblastoma (GBM) is a highly aggressive primary central nervous system tumor with a median survival of 14 months. MGMT (O6-methylguanine-DNA methyltransferase) promoter methylation status is a key biomarker as a prognostic indicator and a predictor of chemotherapy response in GBM. Patients with MGMT methylated disease progress later and survive longer (median survival rate 22 vs. 15 months, respectively) as compared to patients with MGMT unmethylated disease. Patients with GBM undergo an MRI of the brain prior to diagnosis and following surgical resection for radiation therapy planning and ongoing follow-up. There is currently no imaging biomarker for GBM. Studies have attempted to connect MGMT methylation status to MRI imaging appearance to determine if brain MRI can be leveraged to provide MGMT status information non-invasively and more expeditiously. **Methods**: Artificial intelligence (AI) can identify MRI features that are not distinguishable to the human eye and can be linked to MGMT status. We employed the UPenn-GBM dataset patients for whom methylation status was available (*n* = 146), employing a novel radiomic method grounded in hybrid feature selection and weighting to predict MGMT methylation status. **Results**: The best MGMT classification and feature selection result obtained resulted in a mean accuracy rate value of 81.6% utilizing 101 selected features and five-fold cross-validation. **Conclusions**: This compared favorably with similar studies in the literature. Validation with external datasets remains critical to enhance generalizability and propagate robust results while reducing bias. Future directions include multi-channel data integration with radiomic features and deep and ensemble learning methods to improve predictive performance.

## 1. Introduction

Glioblastoma (GBM) is a highly aggressive primary central nervous system tumor with a median survival of 14 months [1]. GBM is typically managed with maximal surgical resection followed by concurrent chemotherapy and radiation therapy [2]. GBM behaved in a treatment-resistant manner, recurring locally with treatment options generally limited upon recurrence. MGMT (O6-methylguanine-DNA methyltransferase) promoter methylation status is a key biomarker as a prognostic indicator and a predictor of chemotherapy response in GBM [3]. Patients with MGMT methylated disease progress later and survive longer (median survival rate 22 vs. 15 months, respectively) as compared to patients with MGMT unmethylated disease. However, the biological mechanisms that underlie this observation are less well understood [4]. MGMT methylation renders aspects of DNA repair inactive, leading to superior response to management. MGMT methylation status is obtained by examining tumor tissue and is typically available to clinicians within 2–3 weeks of surgical resection once pathological analysis has been completed. Current retrospective data in GBM often suffer from sparse annotation for MGMT status, in part due to their later introduction into clinical practice, cost, and sample availability limitations. Patients with GBM undergo an MRI of the brain prior to diagnosis and following surgical resection for radiation therapy planning and ongoing follow-up. The ability to predict MGMT status using MRI could revolutionize preoperative planning for neurosurgeons [5], allow for superior risk stratification [6,7], and help personalize radiation therapy [8]. There is currently no imaging biomarker for GBM. Several studies have attempted to connect MGMT methylation status as a molecular classification to MRI imaging appearance. This would allow the utilization of large-scale MRI brain data for MGMT status annotation. Artificial intelligence (AI) can identify MRI features that are not distinguishable to the human eye and can be linked to MGMT status [9,10,11,12]. The results have been mixed, reaching close to 70% accuracy in recent studies, and are still evolving [13,14,15]. In a recent study [16], a two-phase MGMT Promoter Methylation Prediction (MGMT-PMP) system was employed to extract and combine different types of radiomics features (GLCM, HOG, and LBP) to predict GBM subtype using the BraTS-2021 dataset. In a separate study [17], an area under the curve (AUC) of 0.718 was obtained using a deep learning framework utilizing convolutional neural networks (CNNs) with Bayesian-optimized hyperparameters to predict MGMT status using the RSNA-MICCAI dataset. This present study aims to develop a novel radiomic method grounded in hybrid feature weighting to assess MGMT promoter methylation status.

The key findings of this study are summarized as follows:

- This is the first study that employs a hybrid feature selection and weighting methodology for MGMT status prediction on a large-scale radiomics dataset employing multiparametric MRI (T1, T1-Gd, T2, and T2-FLAIR).
- Radiomics feature selection techniques (filtering (i.e., mRMR) and embedding-based (i.e., LASSO) selection) were employed to refine predictive models and enhance interpretation by identifying the most relevant and low-dimensional imaging features.
- This study focuses on the growing role of AI-driven radiomics in precision oncology, offering a non-invasive alternative to determine MGMT status in patients with GBM.

## 2. Materials and Methods

In this subsection, we give a brief overview of the feature selection and feature weighting scheme utilized, including a general description of the dataset and methodologies used.

### 2.1. Dataset

We employed the dataset from UPenn-GBM (*n* = 671), focusing on the patients for whom methylation status was available (97 methylated and 49 unmethylated), *n* = 146, as downloaded from the TCIA website [18].

### 2.2. Image Preprocessing

The image preprocessing pipeline consists of image registration, brain extraction, and tumor subregion segmentation, following the procedures outlined by Bakas et al. [18].

All multiparameter MRI images are resampled to a 1 mm^3^ isotropic resolution and rigidly registered to the T1-Gd image. To further refine alignment, a greedy diffeomorphic registration method is applied to the rigidly co-registered MRI images [19]. Brain extraction is then performed to remove non-brain tissues, isolating the cerebrum and cerebellum from the skull using the Brain Mask Generator (BrainMaGe) (University of Pennsylvania, Philadelphia, PA, USA), a deep learning (DL) generalizable brain extraction (skull-stripping) tool explicitly implemented for the operations in brain MRI scans was employed [20].

### 2.3. Tumor Segmentation

**Figure 1:**
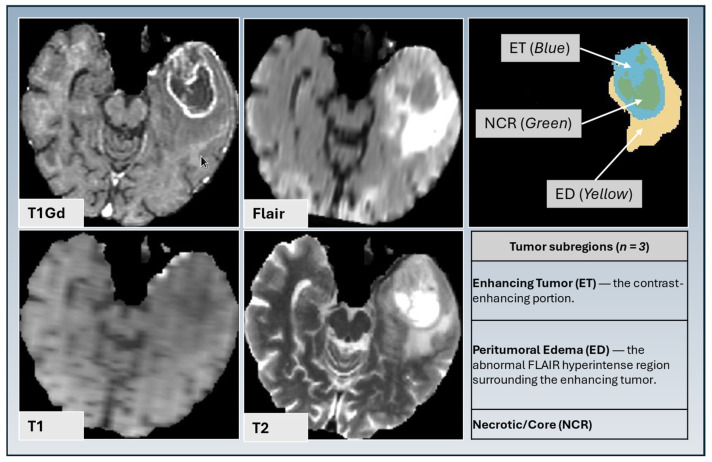
Tumor subregion segmentation based on T1 gadolinium-enhanced MRI, T2, FLAIR, and T1 and T2 sequences, illustrating enhancing tumor (ET) (blue), peritumoral edematous/infiltrated tissue (ED) (yellow), and necrotic tumor core (NCR) (green).

We employed top-performing deep learning methods from the MICCAI BraTS Challenge for tumor segmentation. Tumor subregion segmentation was performed using three top-ranked Brain Tumor Segmentation initiative (BraTS) methods: DeepMedic [21], DeepSCAN [22], and nnU-NET [23] to segment the tumor into three subregions: enhancing tumor (ET), necrotic tumor core (NCR), and peritumoral edematous tissue (ED) (Figure 1). These subregions are segmented using conventional structural MRI modalities, including T1, T1-Gd, T2, and T2-FLAIR weighted images. A label fusion technique integrates outputs from multiple methods to enhance segmentation accuracy. The final segmentation results are manually reviewed by experienced radiologists for validation.

### 2.4. Feature Extraction

Following tumor subregion segmentation, radiomic features are extracted from all MRI modalities to quantify imaging characteristics for each subregion [24]. These features are subsequently used for MGMT promoter methylation status classification.

**Figure 2:**
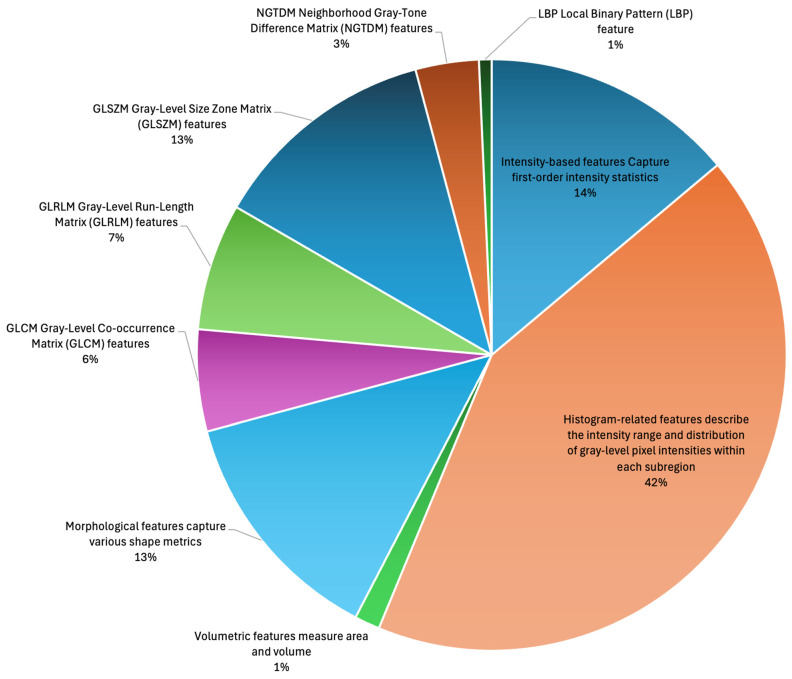
Distribution of extracted radiomic features by feature subtype before feature selection and feature weighting.

One hundred and forty-four radiomic features are computed for each annotated subregion (Table 1, Figure 2). The total number of features is 144 (Table 1) × 11 (Table 2) × 3 (Figure 1), for a total of 4752 features.

### 2.5. Feature Selection

#### Utilized Scheme for Feature Selection and Weighting

**Figure 3:**
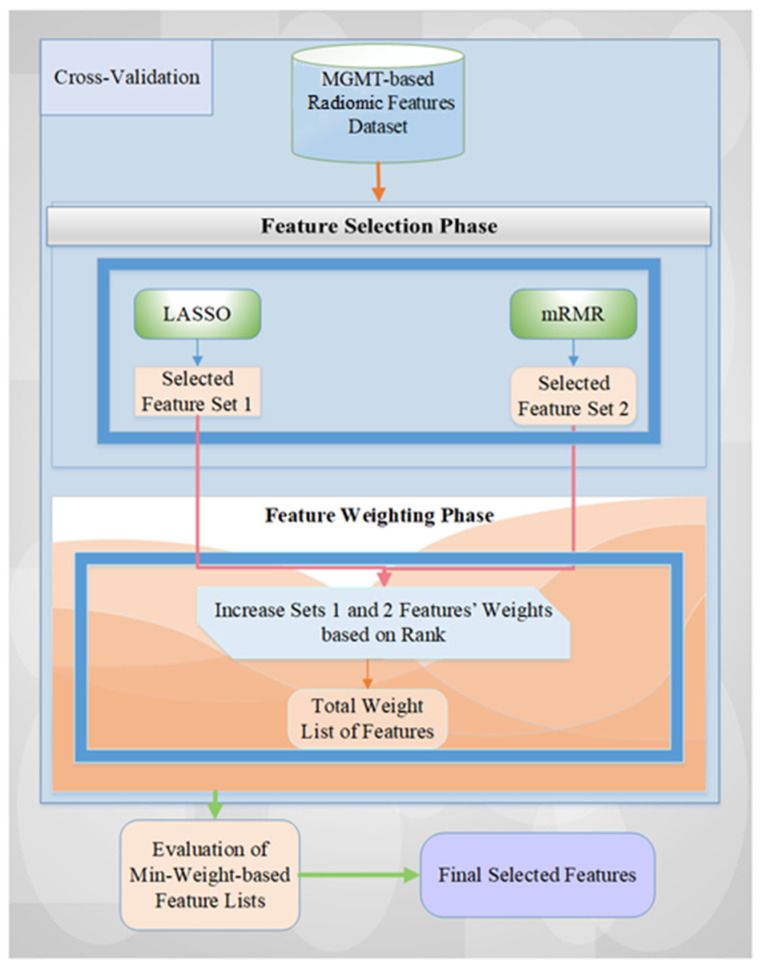
The overview of the employed feature selection and weighted methodology for MGMT promoter status classification based on imaging features.

We generally adopted our previous feature selection and weighted methodology, namely, MGMT ProFWise [25], on the MGMT-based imaging dataset. Our employed architecture includes two essential steps: feature selection (FS) and weighting (FW) [25,26]. These stages combine the popular and efficient two feature subset selection methods: Minimum Redundancy Maximum Relevance (mRMR) and Least Absolute Shrinkage and Selection Operator (LASSO). The detailed schematic diagram is illustrated in Figure 3.

Initially, using a machine learning model and cross-validation method, every radiomic feature obtained from the feature extraction phase is considered a candidate predictor of the feature selection (FS) stage. For each fold, the related feature subsets chosen by the two FS techniques are stored in variables, and the counts of selected features are aggregated based on the weights. Subsequently, the weight-based feature list is reviewed using all weight values, with higher accuracy values indicating more significant importance. Finally, the definitive feature list is created by assessing all weight values and identifying those that yield the highest performance score (i.e., the accuracy rate) with the Support Vector Machine (SVM) model. To investigate this employed methodology, please see the articles from Tasci et al. [25,26,27].

## 3. Results

This section presents the experimental process and explains the performance metric employed. The computational results are also given in the following subsections.

### 3.1. Experimental Process

The proposed methods are implemented in Python 3.9, utilizing the scikit-learn package [28] for LASSO regression and classification and the mRMR package for feature selection [29]. The programs run on a DELL Precision Tower Workstation T7910, equipped with a 20-core 2.20 GHz Xeon CPU and 64 GB of memory, under the Ubuntu 20.04 Linux operating system. After testing various parameter combinations, the optimal performance is achieved using the following settings: a LASSO alpha value of 0.05, a LASSO weighting coefficient of 2.0, an mRMR weighting coefficient of 1.0, and a subset feature selection threshold of 3.0.

### 3.2. Performance Metric

To evaluate the effectiveness of the combined feature selection and weighting method utilized for MGMT classification, we employed the classification accuracy rate (ACC) for the machine learning operations.

For the classification process, the accuracy rate was calculated by taking the total of true positives and true negatives and dividing it by the overall number of instances, which also includes false negatives and false positives [30], as given in Equation (1).

(1) $$\mathrm{ACC}=\frac{\mathrm{TP}+\mathrm{TN}}{\mathrm{TP}+\mathrm{TN}+\mathrm{FP}+\mathrm{FN}}$$

TP, FN, TN, and FP denote the counts of true positives, false negatives, true negatives, and false positives, respectively.

### 3.3. Computational Results

**Figure 4:**
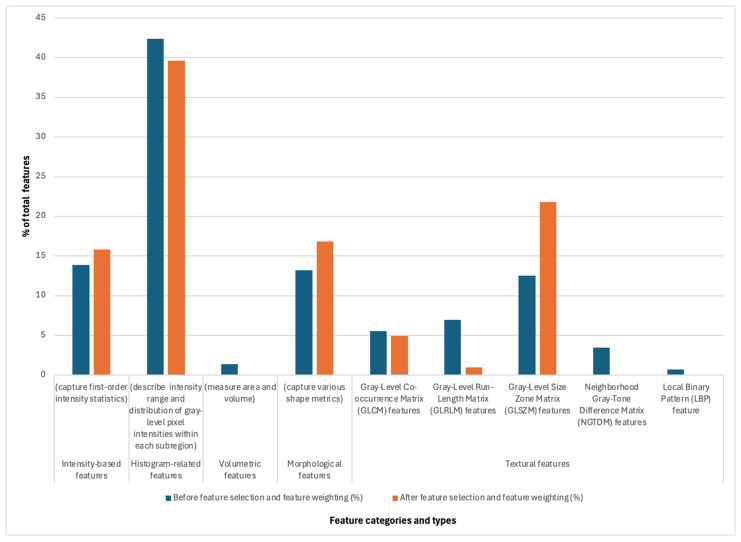
The distribution of extracted radiomic features (%) prior to feature selection and weighting (blue) and after (orange).

In this study, we evaluated the effects of using the no feature selection method, only using individual feature selection (i.e., LASSO and mRMR), and combining the feature selection method with the weighting strategy. The detailed computational results are given in Table 3, along with the accuracy rate of each cross-validation fold. The best result in Table 3 was obtained with a mean accuracy rate value of 81.6% by utilizing 101 selected features (Supplemental File S1) and a minimum weight number of 3, assigning LASSO weight to 2 and mRMR weight to 1. The total number of selected features is one hundred and one, including sixteen intensity features, forty histogram-based features, seventeen morphologic features, five GLCM features, one GLRLM feature, and twenty-two GLSZM features. The distribution of extracted radiomic features (%) prior to feature selection and weighting and after this process is illustrated in Figure 4. When hybrid feature selection and weighting are applied to the imaging dataset, the best possible outcome is a significant performance improvement in the classification accuracy rate. We also calculated the AUC (area under the ROC curve), sensitivity, and specificity results after feature selection and weighting operation. The mean AUC, sensitivity, and specificity values are obtained as 0.760, 0.927, and 0.593, respectively.

### 3.4. Identified Features

The dimensionality reduction process impacted the relative contribution of different feature groups in the final predictive model (Figure 4). Histogram-related features were the most prevalent before and after feature selection at nearly 40% of all features, indicating their strong discriminative power in predicting MGMT status. While their proportion slightly decreased post-selection from 42% to 40%, they remained dominant, suggesting their robust contribution to classification performance. Intensity-based features showed a minor increase from 14% to 16% in representation after feature selection, reflecting their importance in capturing first-order intensity statistics relevant to MGMT methylation status. Volumetric features, initially contributing a small fraction (1.4%), were primarily removed after feature selection, indicating their limited predictive value in this context. Morphological features experienced a notable increase in representation after selection (13% vs. 17%), suggesting that shape-based metrics provide relevant discriminatory information for MGMT status. Textural features exhibited varied effects with Gray-Level Co-occurrence Matrix (GLCM) features decreasing, implying that specific second-order statistical patterns were redundant or less informative. Gray-Level Run-Length Matrix (GLRLM) and Neighborhood Gray-Tone Difference Matrix (NGTDM) features were absent in the final feature set, suggesting limited relevance in the final model prediction. Gray-Level Size Zone Matrix (GLSZM) features substantially increased from 13% to 22%, indicating that zone-size-based textural variations are crucial in the classification. Local Binary Pattern (LBP) features remained at a minimal contribution both before (0.7%) and after feature selection (0%), reinforcing their low relevance in MGMT status prediction. The identified features were ranked according to importance (Supplementary File S1). “DSC_PSR_ET_GLCM_Bins.16_Radius.1_Contrast” was the top identified feature. DSC_PSR refers to Dynamic Susceptibility Contrast (DSC) imaging, and PSR stands for Percent Signal Recovery, a perfusion parameter that can reflect vascular integrity and permeability. ET indicates that the feature is extracted from the enhancing tumor region (the part of the tumor that enhances after contrast injection, which is typically the most aggressive part in glioblastoma), while the GLCM (Gray-Level Co-occurrence Matrix) captures texture or how pixel intensities relate to each other spatially. Bins.16 refers to the number of gray levels the image is quantized into (16 levels), and Radius.“1” indicates the spatial scale examining neighboring voxel interaction within a 1-voxel radius. “Contrast” is a GLCM feature that quantifies the intensity difference between neighboring voxels, with higher contrast indicating sharper transitions between voxels. The second most significant feature was “DSC_ap.rCBV_ET_GLCM_Bins.16_Radius.1_ClusterShade“. This feature is related to relative cerebral blood volume (rCBV) and shares the GLCM, Bins.16, and Radius.1 with the previous feature. “ClusterShade,” the actual feature, indicates irregularity or the difference between more uniform and more heterogeneous textures. Each feature is defined in PyRadiomics [24].

## 4. Discussion

The non-invasive prediction of MGMT status using radiomics has significant clinical implications given that MGMT promoter methylation status is a key prognostic and predictive biomarker in GBM [31,32,33]. The ability to predict MGMT status using imaging as opposed to relying on tumor tissue given sample, cost, and timing constraints has been of critical interest in neuro-oncology [6,14,34]. The current study demonstrates the potential of radiomics-based prediction of GBM, achieving an overall accuracy of 81.6% using feature selection and feature weighting techniques, which aligns favorably with previous studies with accuracy ranging between 70% and 90% [13,15]. The use of feature selection and feature weighting contributed significantly to model optimization, with overall feature reduction from 4752 to 101 features, including 16 intensity features, 40 histogram-based features, 17 morphologic features, 5 GLCM features, 1 GLRLM feature, and 22 GLSZM features. Interestingly, several feature types grew in importance, notably Gray-Level Size Zone Matrix (GLSZM) features, morphological features, and intensity-based features. By contrast, volumetric features, NGTDM, and LBP features were not present in the final feature set, indicating a lesser importance in MGMT prediction overall. Histogram-related features comprised nearly 40% of all features and were sustained with feature selection and weighting. The top identified feature was “DSC_PSR_ET_GLCM_Bins.16_Radius.1_Contrast” and may represent an association with MGMT molecular classification by interrelating contrast enhancement and signal recovery, a perfusion parameter reflecting both vascular integrity and vascular permeability, both clinically relevant and biologically interpretable in GBM. ET indicates that the feature is extracted from the enhancing tumor region, which is generally seen as the most aggressive component of GBM. Interestingly, the GLCM (Gray-Level Co-occurrence Matrix), which examines texture and differences in how pixel intensities relate to each other spatially, may indicate a relationship to tumor heterogeneity. MGMT unmethylated tumors exhibit more significant heterogeneity than MGMT methylated tumors. This aspect may also be reflected in the second most significant feature “DSC_ap.rCBV_ET_GLCM_Bins.16_Radius.1_ClusterShade”, which captures intervoxel heterogeneity and relative cerebral blood volume. This aspect may relate to angiogenesis in GBM, with MGMT unmethylated tumors possibly exhibiting a higher level of angiogenesis as compared to MGMT methylated tumors, as reflected in angiogenesis pathways and glioma subtypes overall [35]. Other studies have also identified second-order statistical textural features, including GLSZM and GLRLM, supporting the critical aspect of capturing heterogeneity related to MGMT methylation status in GBM [36]. In the present study, GLSZM features represented the one feature category with the most increase in representation following feature selection and weighting, in keeping with the importance of this feature set in MGMT prediction, as supported by other studies [36].

One of the critical improvements in the current approach is the application of feature selection and weighting to prioritize features, refining the model’s predictive power, enhancing generalizability, and mitigating overfitting. Many radiomics-based studies suffer from the high-dimensionality problem, where many extracted features can introduce noise and reduce model interpretability. The feature weighting further refined the contribution of each selected feature, ensuring that highly informative features had a more significant impact on classification while reducing the influence of less relevant ones. Our model’s performance is competitive compared to prior studies utilizing conventional machine learning approaches. Previous studies using handcrafted radiomics features combined with machine learning models (SVMs and random forest) have reported AUC values ranging from 0.75 to 0.90 [15,36,37]. Deep learning-based models have achieved slightly higher accuracy in some cases but rely on large training datasets and computational resources, which remains challenging since there is a paucity of large, annotated imaging sets in GBM. Guo et al. [15] proposed a Cascaded Data Processing Network (CDPNet), combining several approaches to estimate MGMT methylation from MRI and achieving an accuracy rate of 70.11% with ten-fold cross-validation. Using deep learning-based methods with different network architectures, another study [13] predicted MGMT methylation status with a mean AUC of 0.6309 with five-fold cross-validation. Shahzad et al. [16] obtained a 96.84 ± 0.09% accuracy rate using different types of radiomics features and ten-fold cross-validation, while Farzana et al. [17] obtained an area under the curve (AUC) of 0.718 using a CNN with Bayesian-optimized hyperparameters.

The present methodology effectively leverages feature selection to optimize computational efficiency by employing three top-performing deep learning methods from the MICCAI BraTS Challenge (DeepMedic [21], DeepSCAN [22], and nnU-NET [23]) to segment the tumor into three subregions. The label fusion technique was employed to improve segmentation precision, providing an advantage by combining multiple segmentation outputs, enhancing accuracy and robustness. In contrast to deep learning-based segmentation models, which can be prone to overfitting or domain-based biases, label fusion integrates results from various methods to establish a consensus segmentation solution, minimizing errors from individual segmentation models. Additionally, it provides computational efficiency, making it suitable for different scenarios where deep learning models demand substantial training data and processing resources [38].

The current study does suffer from several limitations, which include the retrospective nature of the data and the small number of patients with known MGMT status in public data, including, in this case, the UPenn-GBM/TCIA data, where MGMT status is known in 22% (*n* = 146) of the cohort. While the predictive model exhibited a robust classification performance when applied to the dataset, it requires validation in independent datasets. Validation efforts in several studies have been hampered by the heterogeneity of MRI protocols across institutions, limiting generalizability. We performed five-fold cross-validation to demonstrate model stability and strengthen the robustness and generalizability of the results. Additional explanations of the limitations and possible solutions can be obtained from [39]. Future directions include using deep learning-based radiomics approaches and integrating clinical, proteomic, and metabolomic data to improve predictive accuracy further.

## 5. Conclusions

In the current study, we proposed a novel method for MGMT methylation status classification in GBM by employing radiomic features in MRI scans as a non-invasive approach for obtaining MGMT promoter methylation status. The approach achieved high accuracy (81.6%) using feature selection and weighting with interpretable performance improvement superior or comparable to contemporary data, suggesting a promising ability to predict MGMT status using standard brain MRI. While the current results require further validation in independent datasets, the method presented here could significantly impact the clinical management and survival of GBM patients. Our key findings demonstrate why external validation is a fundamental step and one of the limitations of our study. This can be useful for improving the results and ensuring model effectiveness for real-world implications (e.g., reliability or reducing biased outcomes) of the research. As MGMT methylation data annotation is growing, retrospective imaging where MGMT status was obtained may allow for further analysis and examination of the mechanistic interplay between omic signals, imaging appearance of GBM, and outcomes.

## Figures and Tables

**Table 1:** Extracted radiomic features for each annotated subregion. Bold text shows the feature-related titles.

Feature Type (Category)	Feature Description	Radiomic Features (*n* = 144)
**Intensity-based features**	Capture first-order intensity statistics	20
**Histogram-related features**	Describe the intensity range and distribution of gray-level pixel intensities within each subregion	61
**Volumetric features**	Measure area and volume	2
**Morphological features**	Capture various shape metrics	19
**Texture descriptors**	Represent local variation and spatial dependence of image intensities	
	Gray-Level Co-occurrence Matrix (GLCM) features	8
	Gray-Level Run-Length Matrix (GLRLM) features	10
	Gray-Level Size Zone Matrix (GLSZM) features	18
	Neighborhood Gray-Tone Difference Matrix (NGTDM) features	5
	Local Binary Pattern (LBP) feature	1

**Table 2:** Imaging sequences analyzed.

Imaging Sequences (*n* = 11)
T1 pre-contrast
T1 post-contrast (T1Gd)
T2 weighted
FLAIR
DSC (perfusion)—rCBV map
DSC—rCBF map
DSC—PSR map
DTI—FA map
DTI—MD map
ADC map (from DWI)
SWI (susceptibility-weighted imaging)

T1Gd (T1 Gadolinium), FLAIR (Fluid-Attenuated Inversion Recovery), DSC (Dynamic susceptibility contrast), rCBV (relative cerebral blood volume), rCBF (regional cerebral blood flow), PSR (percentage signal recovery), FA (fractional anisotropy), MD (mean diffusivity), ADC (apparent diffusion coefficient), DWI (diffusion weighten imaging), SWI (susceptibility weighted imaging).

**Table 3:** Performance results prior to feature selection (FS) with LASSO, mRMR, and post-feature weighting.

	Fold 1	Fold 2	Fold 3	Fold 4	Fold 5	Mean
**Before FS**	0.667	0.69	0.655	0.655	0.586	0.651
**LASSO**	0.533	0.724	0.655	0.655	0.552	0.624
**mRMR**	0.667	0.69	0.69	0.655	0.448	0.630
**Feature Weighting**	0.767	0.862	0.828	0.931	0.69	0.816

## Data Availability

Publicly available datasets from the University of Pennsylvania glioblastoma (UPenn-GBM) cohort were analyzed in this study. This data can be found here: [https://www.cancerimagingarchive.net/collection/upenn-gbm/ (accessed on 15 May 2025)].

## Supplementary Materials

The following supporting information can be downloaded at https://www.mdpi.com/article/10.3390/diagnostics15101292/s1, Supplementary File S1: Identified features and weighting of each feature after feature selection and weighting.

## Abbreviations

AI	Artificial Intelligence
AUC	Area Under the ROC Curve
BraTS	Brain Tumor Segmentation
CaPTk	Cancer Imaging Phenomics Toolkit
CET	Contrast-Enhancing Tumor
CNS	Central Nervous System
DSC	Dynamic Susceptibility Contrast
ED	Peritumoral Edema
ET	Enhancing Tumor
FLAIR	Fluid-Attenuated Inversion Recovery
GBM	Glioblastoma
LASSO	Least Absolute Shrinkage and Selection Operator
MGMT	O6-Methylguanine-DNA Methyltransferase
MRMR	Minimum Redundancy Maximum Relevance
MRI	Magnetic Resonance Imaging
NCR	Necrotic Tumor Core
PSR	Percent Signal Recovery
RBF	Radial Basis Function
rCBV	Relative Cerebral Blood Volume
SVM	Support Vector Machine

## References

[1] Louis D.N., Perry A., Wesseling P., Brat D.J., Cree I.A., Figarella-Branger D., Hawkins C., Ng H.K., Pfister S.M., Reifenberger G. (2021). The 2021 WHO Classification of Tumors of the Central Nervous System: A summary. Neuro Oncol..

[2] Stupp R., Mason W.P., van den Bent M.J., Weller M., Fisher B., Taphoorn M.J., Belanger K., Brandes A.A., Marosi C., Bogdahn U. (2005). Radiotherapy plus concomitant and adjuvant temozolomide for glioblastoma. N. Engl. J. Med..

[3] Butler M., Pongor L., Su Y.T., Xi L., Raffeld M., Quezado M., Trepel J., Aldape K., Pommier Y., Wu J. (2020). MGMT Status as a Clinical Biomarker in Glioblastoma. Trends Cancer.

[4] Fang Q. (2024). The Versatile Attributes of MGMT: Its Repair Mechanism, Crosstalk with Other DNA Repair Pathways, and Its Role in Cancer. Cancers.

[5] Katsigiannis S., Grau S., Krischek B., Er K., Pintea B., Goldbrunner R., Stavrinou P. (2021). MGMT-Positive vs MGMT-Negative Patients With Glioblastoma: Identification of Prognostic Factors and Resection Threshold. Neurosurgery.

[6] Samartha M.V.S., Dubey N.K., Jena B., Maheswar G., Lo W.C., Saxena S. (2024). AI-driven estimation of O6 methylguanine-DNA-methyltransferase (MGMT) promoter methylation in glioblastoma patients: A systematic review with bias analysis. J. Cancer Res. Clin. Oncol..

[7] Bell E.H., Pugh S.L., McElroy J.P., Gilbert M.R., Mehta M., Klimowicz A.C., Magliocco A., Bredel M., Robe P., Grosu A.L. (2017). Molecular-Based Recursive Partitioning Analysis Model for Glioblastoma in the Temozolomide Era: A Correlative Analysis Based on NRG Oncology RTOG 0525. JAMA Oncol..

[8] Yun H.S., Kramp T.R., Palanichamy K., Tofilon P.J., Camphausen K. (2024). MGMT inhibition regulates radioresponse in GBM, GSC, and melanoma. Sci. Rep..

[9] Smits M., van den Bent M.J. (2017). Imaging Correlates of Adult Glioma Genotypes. Radiology.

[10] Han Y., Yan L.F., Wang X.B., Sun Y.Z., Zhang X., Liu Z.C., Nan H.Y., Hu Y.C., Yang Y., Zhang J. (2018). Structural and advanced imaging in predicting MGMT promoter methylation of primary glioblastoma: A region of interest based analysis. BMC Cancer.

[11] Drabycz S., Roldán G., de Robles P., Adler D., McIntyre J.B., Magliocco A.M., Cairncross J.G., Mitchell J.R. (2010). An analysis of image texture, tumor location, and MGMT promoter methylation in glioblastoma using magnetic resonance imaging. Neuroimage.

[12] Hong E.K., Choi S.H., Shin D.J., Jo S.W., Yoo R.E., Kang K.M., Yun T.J., Kim J.H., Sohn C.H., Park S.H. (2018). Radiogenomics correlation between MR imaging features and major genetic profiles in glioblastoma. Eur. Radiol..

[13] Saeed N., Ridzuan M., Alasmawi H., Sobirov I., Yaqub M. (2023). MGMT promoter methylation status prediction using MRI scans? An extensive experimental evaluation of deep learning models. Med. Image Anal..

[14] Zwanenburg A., Vallieres M., Abdalah M.A., Aerts H., Andrearczyk V., Apte A., Ashrafinia S., Bakas S., Beukinga R.J., Boellaard R. (2020). The Image Biomarker Standardization Initiative: Standardized Quantitative Radiomics for High-Throughput Image-based Phenotyping. Radiology.

[15] Guo J., Yu F., Nasrallah M.P., Davatzikos C. CDPNet: A radiomic feature learning method with epigenetic application to estimating MGMT promoter methylation status in glioblastoma. Proceedings of the SPIE—The International Society for Optical Engineering.

[16] Qureshi S.A., Hussain L., Ibrar U., Alabdulkreem E., Nour M.K., Alqahtani M.S., Nafie F.M., Mohamed A., Mohammed G.P., Duong T.Q. (2023). Radiogenomic classification for MGMT promoter methylation status using multi-omics fused feature space for least invasive diagnosis through mpMRI scans. Sci. Rep..

[17] Farzana W., Temtam A.G., Shboul Z.A., Rahman M.M., Sadique M.S., Iftekharuddin K.M. (2021). Radiogenomic prediction of MGMT using deep learning with Bayesian optimized hyperparameters. Proceedings of the International MICCAI Brainlesion Workshop.

[18] Bakas S., Sako C., Akbari H., Bilello M., Sotiras A., Shukla G., Rudie J.D., Santamaría N.F., Kazerooni A.F., Pati S. (2022). The University of Pennsylvania glioblastoma (UPenn-GBM) cohort: Advanced MRI, clinical, genomics, & radiomics. Sci. Data.

[19] Joshi S., Davis B., Jomier M., Gerig G. (2004). Unbiased diffeomorphic atlas construction for computational anatomy. Neuroimage.

[20] Thakur S., Doshi J., Pati S., Rathore S., Sako C., Bilello M., Ha S.M., Shukla G., Flanders A., Kotrotsou A. (2020). Brain extraction on MRI scans in presence of diffuse glioma: Multi-institutional performance evaluation of deep learning methods and robust modality-agnostic training. NeuroImage.

[21] Kamnitsas K., Ledig C., Newcombe V.F.J., Simpson J.P., Kane A.D., Menon D.K., Rueckert D., Glocker B. (2017). Efficient multi-scale 3D CNN with fully connected CRF for accurate brain lesion segmentation. Med. Image Anal..

[22] McKinley R., Rebsamen M., Daetwyler K., Meier R., Radojewski P., Wiest R. Uncertainty-driven refinement of tumor-core segmentation using 3D-to-2D networks with label uncertainty. Proceedings of the BrainLes@MICCAI.

[23] Isensee F., Jäger P.F., Full P.M., Vollmuth P., Maier-Hein K.H. (2021). nnU-Net for Brain Tumor Segmentation.

[24] van Griethuysen J.J.M., Fedorov A., Parmar C., Hosny A., Aucoin N., Narayan V., Beets-Tan R.G.H., Fillion-Robin J.C., Pieper S., Aerts H. (2017). Computational Radiomics System to Decode the Radiographic Phenotype. Cancer Res..

[25] Tasci E., Shah Y., Jagasia S., Zhuge Y., Shephard J., Johnson M.O., Elemento O., Joyce T., Chappidi S., Cooley Zgela T. (2024). MGMT ProFWise: Unlocking a New Application for Combined Feature Selection and the Rank-Based Weighting Method to Link MGMT Methylation Status to Serum Protein Expression in Patients with Glioblastoma. Int. J. Mol. Sci..

[26] Tasci E., Jagasia S., Zhuge Y., Sproull M., Cooley Zgela T., Mackey M., Camphausen K., Krauze A.V. (2023). RadWise: A Rank-Based Hybrid Feature Weighting and Selection Method for Proteomic Categorization of Chemoirradiation in Patients with Glioblastoma. Cancers.

[27] Tasci E., Popa M., Zhuge Y., Chappidi S., Zhang L., Zgela T.C., Sproull M., Mackey M., Kates H.R., Garrett T.J. (2024). MetaWise: Combined Feature Selection and Weighting Method to Link the Serum Metabolome to Treatment Response and Survival in Glioblastoma. Int. J. Mol. Sci..

[28] Scikit-Learn. https://scikit-learn.org/stable/.

[29] mRMR Feature Selection. https://github.com/smazzanti/mrmr.

[30] Fawcett T. (2006). An introduction to ROC analysis. Pattern Recognit. Lett..

[31] Leske H., Camenisch Gross U., Hofer S., Neidert M.C., Leske S., Weller M., Lehnick D., Rushing E.J. (2023). MGMT methylation pattern of long-term and short-term survivors of glioblastoma reveals CpGs of the enhancer region to be of high prognostic value. Acta Neuropathol. Commun..

[32] Mansouri A., Hachem L.D., Mansouri S., Nassiri F., Laperriere N.J., Xia D., Lindeman N.I., Wen P.Y., Chakravarti A., Mehta M.P. (2019). MGMT promoter methylation status testing to guide therapy for glioblastoma: Refining the approach based on emerging evidence and current challenges. Neuro Oncol..

[33] Hegi M.E., Diserens A.C., Gorlia T., Hamou M.F., de Tribolet N., Weller M., Kros J.M., Hainfellner J.A., Mason W., Mariani L. (2005). MGMT gene silencing and benefit from temozolomide in glioblastoma. N. Engl. J. Med..

[34] Tseng C.L., Zeng K.L., Mellon E.A., Soltys S.G., Ruschin M., Lau A.Z., Lutsik N.S., Chan R.W., Detsky J., Stewart J. (2024). Evolving concepts in margin strategies and adaptive radiotherapy for glioblastoma: A new future is on the horizon. Neuro Oncol..

[35] Zhang Q., Guo Y.X., Zhang W.L., Lian H.Y., Iranzad N., Wang E., Li Y.C., Tong H.C., Li L.Y., Dong L.Y. (2022). Intra-tumoral angiogenesis correlates with immune features and prognosis in glioma. Aging.

[36] Do D.T., Yang M.-R., Lam L.H.T., Le N.Q.K., Wu Y.-W. (2022). Improving MGMT methylation status prediction of glioblastoma through optimizing radiomics features using genetic algorithm-based machine learning approach. Sci. Rep..

[37] Qian J., Herman M.G., Brinkmann D.H., Laack N.N., Kemp B.J., Hunt C.H., Lowe V., Pafundi D.H. (2020). Prediction of MGMT Status for Glioblastoma Patients Using Radiomics Feature Extraction From (18)F-DOPA-PET Imaging. Int. J. Radiat. Oncol. Biol. Phys..

[38] Wang H., Yushkevich P.A. (2013). Multi-atlas segmentation with joint label fusion and corrective learning—An open source implementation. Front. Neuroinform..

[39] Bonada M., Rossi L.F., Carone G., Panico F., Cofano F., Fiaschi P., Garbossa D., Di Meco F., Bianconi A. (2024). Deep Learning for MRI segmentation and molecular subtyping in glioblastoma: Critical aspects from an emerging field. Biomedicines.

